# Gene therapy for epilepsy: An emerging, promising approach for a serious neurological disorder

**DOI:** 10.1111/joim.70059

**Published:** 2025-12-09

**Authors:** Marco Ledri, Merab Kokaia

**Affiliations:** ^1^ Epilepsy Center, Department of Clinical Sciences, Faculty of Medicine Lund University Lund Sweden

**Keywords:** chemogenetics, epilepsy, gene therapy, optogenetics, targeted therapy, viral vectors

## Abstract

Gene therapy is emerging as a groundbreaking strategy for treating epilepsy, offering new hope to patients who do not respond to conventional medications. Despite advancements in anti‐seizure treatments, nearly 30%–40% of individuals with epilepsy continue to experience uncontrolled seizures, highlighting the urgent need for more effective and long‐lasting solutions. By addressing the underlying causes of epilepsy at the genetic level, gene therapy represents a paradigm shift in treatment. Two key approaches are being explored: (1) activating or supplementing beneficial genes to suppress seizures and (2) silencing harmful genes or pathways that contribute to epilepsy. To achieve these objectives, viral vectors, such as adeno‐associated viruses and lentiviruses, have shown promise in delivering targeted genetic interventions. In parallel, cutting‐edge techniques such as optogenetics, chemogenetics, and clustered regularly interspaced short palindromic repeat‐based gene editing are enhancing the precision of these therapies, enabling greater control over neuronal activity. However, significant challenges exist, including ensuring safe and efficient gene delivery, maintaining long‐term therapeutic effects, and mitigating potential side effects. This review examines recent developments in gene therapy for epilepsy, assessing its potential to deliver targeted, long‐lasting treatments for drug‐resistant epilepsy. By examining current strategies, therapeutic targets, and emerging technologies, it provides insights into the promising future of gene therapy as a transformative tool in epilepsy treatment and summarizes current clinical trials utilizing gene and cell therapy technologies for epilepsy.

## Graphical abstract

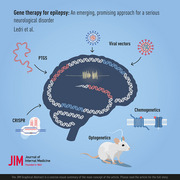

## Introduction

Gene therapy has emerged as a promising frontier in the treatment of numerous neurological disorders, with epilepsy standing as a particularly challenging condition due to its complex pathophysiology and the limitations of existing therapeutic strategies. Affecting approximately 50 million individuals, epilepsy is characterized by recurrent, unprovoked seizures resulting from abnormal, excessive, and hypersynchronous neuronal activity. Despite substantial progress in pharmacological interventions, 30%–40% of patients continue to experience seizures that are refractory to anti‐seizure medications [1, 2]. This clinical reality demonstrates the necessity for innovative therapies capable of addressing the underlying mechanisms of epilepsy instead of merely managing its symptoms pharmacologically. Advances in molecular biology, neuroscience, and bioengineering have driven the development of gene therapy strategies designed to modulate neuronal excitability, restore normal brain function, and mitigate the hallmarks of epilepsy. As a result, gene therapy holds potential as a targeted, long‐lasting, and personalized approach to epilepsy treatment.

Current gene therapy strategies can be categorized into two broad approaches: (i) gene activation or supplementation methods, which involve upregulating seizure‐suppressing genes or replacing mutated genes with functional copies using viral vector‐mediated gene delivery and (ii) gene suppression techniques, such as microRNA (miRNA)‐based posttranscriptional silencing and clustered regularly interspaced short palindromic repeat (CRISPR) interference, which reduce the expression of pro‐epileptogenic genes. These distinct but complementary approaches provide innovative therapeutic solutions tailored to specific forms of epilepsy, particularly monogenic epilepsies and focal epilepsies. In addition, gene therapy strategies can be designed to either simply control symptoms or to modify disease processes and potentially affect comorbidities such as cognitive decline, but also depression, anxiety, and dementia.

Identifying therapeutic targets such as neuropeptides, ion channels, and growth factors has enabled the design of gene therapies tailored to epilepsy's molecular mechanisms. Furthermore, advanced techniques such as optogenetics, chemogenetics, and CRISPR‐based gene editing aim to enhance precision and enable spatiotemporal control over neural activity, advancing preclinical research to promising new levels. Key research efforts have also emphasized exploring the translational and clinical potential of gene therapy, addressing critical challenges such as efficient delivery, sustained expression, and safety.

By integrating the main findings from literature, including preclinical studies and clinical trial data, this review examines the central research question: Can gene therapy provide targeted, effective, and durable solutions for epilepsy, particularly for drug‐resistant cases? Through this analysis, the discussed methodologies and emerging advancements aim to evaluate the feasibility of gene therapy as a novel therapeutic paradigm for epilepsy. To present these findings systematically, the review is structured into several sections. We first provide an analysis of gene therapy strategies and the most common delivery methods, evaluating their respective benefits and challenges (“Gene delivery methods” section). We then explore therapeutic targets, examining the molecular candidates for gene therapy interventions and their roles in epilepsy treatment (“Therapeutic targets” section). Next, we examine advanced strategies such as optogenetics, chemogenetics, and CRISPR‐based gene editing, assessing their role in precision therapy and emerging applications (“Advanced gene therapy applications” section). Finally, we synthesize the findings, describe current clinical trials, and reflect upon translational challenges, key outcomes, and strategic priorities for future research and development (“Clinical trials and current status” section).

## Gene delivery methods

### Viral vectors

The potential of gene therapy for epilepsy relies heavily on viral vectors. This section will examine the distinct characteristics, advantages, and challenges posed by the most widely used vectors for gene therapy, adeno‐associated viruses (AAVs) and lentiviral vectors (LVs).

#### Adeno‐associated virus

AAVs are recognized as one of the most effective vectors for gene therapy, not only in the context of epilepsy, due to their unique characteristics. AAVs are nonpathogenic, and they generally do not integrate into the host genome, which minimizes the risk of insertional mutagenesis, a major safety concern in gene therapy [3]. AAVs are capable of transducing nondividing cells, ensuring their applicability in the context of chronic epilepsy, where neuronal targets are predominant. Their ability to sustain transgene expression for extended periods, demonstrated by preclinical studies and emerging human data showing stable expression for over 10 years, underscores their relevance for lifelong conditions such as epilepsy [4]. These unique attributes position AAVs as one of the leading tools for gene delivery in epilepsy gene therapy.

The favorable immune profile of AAV vectors further strengthens their application in treating epilepsy and other neurological disorders. Preclinical and clinical studies have found that AAV vectors elicit a weaker immune response compared to other viral vectors such as lentiviruses (LVs) or herpes simplex viruses [5]. Their minimal immunogenicity becomes particularly critical in scenarios potentially requiring repetitive treatments, as for refractory epilepsy, as lower immune reactivity reduces complications during subsequent administrations. However, preexisting immunity to AAVs in humans is a significant challenge for AAV‐based gene therapy. Many people have been naturally exposed to AAVs, leading to the development of neutralizing antibodies (NAbs) and T‐cell responses. The prevalence of AAV immunity varies by serotype and population, with AAV2 showing the highest rates (30%–80% of adults), whereas AAV8 and AAV9 have slightly lower prevalence (30%–60%) [6]. This immunity can reduce the effectiveness of gene therapy by neutralizing viral vectors before they can deliver their genetic payload or by triggering immune responses that eliminate transduced cells. Humoral immunity—driven by NAbs—prevents AAV entry into cells, whereas cellular immunity—particularly cytotoxic T‐cells—can recognize and destroy AAV‐infected cells. To overcome these challenges, strategies such as serotype switching, capsid engineering, plasma exchange, immunosuppression, or increasing vector doses can be employed, though higher doses raise other safety concerns. In clinical trials, patients are often screened for AAV antibodies to determine eligibility, whereas next‐generation AAV vectors with reduced immunogenicity are being developed to enhance therapeutic efficacy.

Another advantageous characteristic of AAVs is their natural neuronal tropism, which can be further influenced by capsid engineering. Capsid modifications allow for the development of AAV serotypes tailored to target specific brain regions or cell types, enhancing treatment precision [7]. Alterations in the capsid proteins’ primary sequences can direct AAVs to specific neuronal or glial populations, enabling more effective and specific gene delivery [8]. In addition, capsid‐modified serotypes such as PHP.eB allow widespread CNS transduction after intravenous administration, enabling access to larger brain areas with single vector administration [9]. However, their efficiency seems to be variable and dependent on host genetic background [10], and their effectiveness in humans is still uncertain. Other innovative approaches, such as transferrin receptor 1‐targeting capsids, have significantly improved BBB penetration, achieving extensive transduction of both neurons and astrocytes across the CNS [11]. These capsids exploit receptor‐mediated endocytosis, enhancing their ability to bypass the BBB and reach epileptogenic tissues effectively. This advancement not only supports noninvasive administration methods but also broadens the therapeutic scope for systemic or generalized epilepsy, which requires widespread CNS transduction [11]. However, the variability in capsid performance across different species underscores the need for thorough preclinical testing before transitioning to clinical trials.

Despite their promising therapeutic potential, systemic AAV delivery faces notable translational challenges, including dose‐dependent toxicity. High‐dose systemic AAV administration is associated with hepatotoxicity and neurotoxicity, which might limit its use for systemic applications [7, 12]. Localized delivery methods, such as intraparenchymal injections, offer an effective alternative by minimizing systemic exposure and ensuring targeted action within epileptogenic zones. However, the coverage of larger brain areas, as required for some epilepsy forms in the human brain, would necessitate multiple injections, increasing surgery‐associated risks. Regulatory frameworks emphasize the importance of addressing these safety concerns through robust preclinical and clinical evaluations, highlighting the critical need for careful optimization of dosage and delivery methods.

One of the most studied serotypes, AAV9, has demonstrated exceptional efficacy in CNS applications. It enables widespread transgene expression with minimal immune reactions, even when delivered intravenously [13]. AAV9's strong CNS tropism supports its utility for treating generalized epilepsy, as it enables widespread transduction across the brain regions implicated in the disease. Studies showing its consistent safety profile at therapeutic doses further strengthen its clinical relevance [14, 15]. Moreover, its ability to transduce both neurons and glia makes it a versatile tool for developing combinatorial therapies addressing multiple epileptogenic pathways [13]. Nonetheless, further research is required to fully assess its long‐term safety in humans, particularly in the context of systemic delivery.

The choice of capsid and route of administration is therefore crucial when translating findings from animal models to clinical applications. However, the variety of options available opens avenues for personalized treatments, tailored to both the patient's seizure profile and the specific characteristics of different epilepsy syndromes.

Despite all the mentioned benefits, AAVs are limited by their small DNA packaging capacity, which restricts the size of therapeutic genes to approximately 4.5–5.2 kb. This limitation contrasts with other viral vectors, such as LVs, which can accommodate larger genetic payloads of up to approximately 9 kb. Strategies to address AAV's packaging constraints include the development of compact genetic payloads, such as miRNA and gene fragments, as well as dual‐vector systems, where complementary AAVs deliver separate components of a larger therapeutic gene to achieve functional expression [16, 17]. Although these strategies offer promising solutions to circumvent packaging limitations, they also add complexity to therapy development, necessitating further optimization to balance efficacy and safety. Additionally, the need for administering multiple viral vectors introduces regulatory challenges, complicating the approval and clearance process.

On a positive note, AAV‐mediated transduction exhibits remarkable efficiency in both neurons and astrocytes. This dual targeting capability might be particularly advantageous for epilepsy therapy, as it allows for modulation of excitatory and inhibitory neurotransmission as well as glial regulation of neural circuits. Targeting both neurons and astrocytes expands the therapeutic potential of gene therapy, as it allows consideration of not only neuronal hyperexcitability and network dysfunction but also significant glial impairments in the multifaceted pathophysiology of epilepsy [8].

#### Lentivirus

LVs represent another pivotal tool in gene therapy for epilepsy. Their ability to package up to 9 kb of DNA makes them well‐suited for incorporating larger or more intricate genes, providing significant advantages when designing and evaluating more advanced therapeutic strategies. Unlike AAVs, LVs can deliver multiple transgenes simultaneously. This capability is crucial for targeting multiple pathways in epilepsy, for example, by combining genes that enhance inhibitory mechanisms while concurrently suppressing excitatory pathways. This combinatorial approach has the potential to improve therapeutic outcomes by addressing the complex nature of epilepsy. However, although the larger packaging capacity is advantageous, the challenge of achieving effective and uniform distribution within the CNS remains a significant hurdle, necessitating innovative strategies to enhance LV delivery. LVs are less effective for CNS transduction in broader regions of the brain tissue due to their larger size and limited diffusion, and they carry different safety concerns.

Significant advancements in LV design have aimed to enhance safety while maintaining efficacy. Traditional LVs pose concerns regarding integration into the host genome, which carries risks of insertional mutagenesis [18]. However, recent innovations in non‐integrating LV systems and self‐inactivating (SIN) vectors have substantially reduced these risks, making them safer options for therapeutic applications [19]. These vectors avoid permanent genomic integration while maintaining sustained transgene expression, effectively mitigating the risk of insertional mutagenesis [19]. Innovative designs such as SIN vectors have further refined the safety profile of LVs by eliminating promoter activity in the vector's long terminal repeats, reducing genotoxicity without compromising therapeutic efficacy [20]. These advancements allow stable transfer of larger genes without compromising neuronal function. Such improvements in LV safety hold great promise for clinical translation, yet further studies are crucial to rigorously validate these protocols across diverse conditions.

Despite their advantages, the limited diffusion of LVs through the brain's extracellular matrix presents a significant challenge. This limitation restricts their efficiency in targeting larger or widely distributed epileptic regions. Approaches such as modifying the vector envelope through pseudotyping with glycoproteins have been proposed to enhance diffusion and penetration into neuronal networks, improving therapeutic coverage [21, 22]. Nanotechnology‐based strategies also offer promise, with the coupling of LVs to nanoparticles allowing for enhanced distribution and diffusion, potentially enabling CNS coverage of larger brain areas [23]. However, these solutions add complexity and may introduce new variables, such as nanoparticle‐related toxicity, which require thorough investigation before clinical application.

Clinical translation of LV‐based therapies faces several additional obstacles, including immune responses, difficulties with targeted delivery, and long‐term safety concerns. Immune responses against LVs remain a significant obstacle to widespread clinical adoption [24, 25]. Strategies such as transient immunosuppression during administration or the development of immune‐evasive capsids could mitigate these challenges and improve the likelihood of successful clinical outcomes [26].

## Therapeutic targets

The intricate interplay of excitatory and inhibitory mechanisms is fundamental to normal brain function, yet this equilibrium is often disrupted in epilepsy. Like certain conventional pharmacological therapies, gene therapy aims to restore neuronal network stability to reduce seizure risk. The following sections explore innovative strategies designed to rectify imbalances in neuronal activity and improve overall therapeutic outcomes by modulating key therapeutic targets such as ion channels, neuropeptides, and growth factors.

### Ion channels

Ion channels represent a critical therapeutic target for epilepsy, given their central role in controlling and modulating neuronal excitability. Among the most extensively studied targets are potassium channels, particularly the Kv1.1 channel, whose overexpression has demonstrated significant potential in reducing seizure frequency [27, 28, 29, 30]. The underlying mechanism involves stabilizing the resting membrane potential, thereby limiting excessive neuronal firing rates in epileptogenic brain regions. By stabilizing a hyperpolarized state, Kv1.1 channels reduce hyperexcitability, offering a valuable strategy to manage epilepsy. Cell‐specific promoters, such as CaMKIIa, enhance the precision of this approach by ensuring that transgene expression is predominantly confined to excitatory neurons. This targeted strategy minimizes the risks associated with unintended expression in inhibitory neurons or glial populations, thereby improving both efficacy and safety [28, 29]. Overexpression of Kv1.1 was effective in both focal neocortical [28, 29] and temporal lobe epilepsy (TLE) models [29, 30]. This research also suggests that Kv1.1 overexpression might be relevant not only in epilepsy but also in treating conditions such as chronic pain, where neuronal hyperexcitability is a pathological feature.

Manipulating sodium channels has also emerged as a promising avenue for epilepsy treatment, particularly targeting Nav1.1 channels (coded by the *SCN1A* gene) in inhibitory neurons. The upregulation of Nav1.1 in interneurons would enhance gamma‐aminobutyric acid (GABA) release and restore the critical balance between excitatory and inhibitory signaling in neuronal networks. However, due to the size of the Nav1.1 protein—exceeding 260 kDa, and therefore the capacity of AAV vectors—strategies for its upregulation have relied on other vector types or on transcriptional activation. Adenoviral‐mediated upregulation of *SCN1A* in GABAergic neurons significantly reduced sudden death and alleviated the epileptic phenotype in a Dravet syndrome mouse model when the vector was administered at 5 weeks of age [31]. Similarly, canine adenovirus type 2 mediated overexpression of *SCN1A* reduced the occurrence of epileptic spikes, provided protection from thermally induced seizures, and improved Dravet syndrome comorbidities [32]. It has to be noted, however, that transgene expression from adenoviral vectors typically has a limited duration, and therefore gene therapies using these vectors need to potentially be repeated multiple times to provide long‐term symptomatic benefit. Transcriptional activation of *SCN1A* by either engineered transcription factors or CRISPRa has also demonstrated therapeutic potential in Dravet syndrome [33, 34, 35]. Importantly, some of these approaches have also been shown to have widespread expression and were well tolerated in nonhuman primates [33], supporting potential clinical development of such therapies.

*N*‐methyl‐d‐aspartate receptors (NMDARs) play a critical role in glutamatergic excitatory synaptic transmission and plasticity in the CNS. NMDARs modulators have long been considered as potential treatments for several psychiatric disorders, including schizophrenia and depression, and small NMDA inhibitors have been recently developed to target Alzheimer's disease. In the context of epilepsy, given the critical role of NMDARs in hyperexcitability and excitotoxicity, several studies have investigated the potential of knocking down their expression as a therapeutic strategy. Using an AAV‐mediated delivery of an NR1 subunit targeting antisense sequence in the temporal cortex resulted in increased threshold for electrical induction of seizures [36]. Similar effects were observed when shRNAs designed to knock down NR1 subunits were delivered by AAV in the hippocampus, which protected rats from local kainic acid‐induced seizures [37]. Additionally, seizure protection was observed when miRNA‐mediated downregulation of GluK2‐containing kainate receptors was applied in the hippocampus by AAV [38]. However, it is important to note that while NMDAR downregulation provides seizure protection, it also leads to impairments in hippocampal‐dependent learning and neurogenesis, which could limit the clinical viability of these strategies.

In parallel to decreasing excitation via downregulation of excitatory receptors, increasing inhibition by overexpressing GABA receptors has also been explored. GABA_A_ receptors are responsible for GABA‐mediated phasic inhibition in the CNS, and some of their subunits are consistently downregulated in animal models of epilepsy and in brain tissue from patients. Therefore, a strategy aimed at restoring their expression could theoretically reestablish inhibitory balance. An approach utilizing AAV vectors carrying the α1 subunit transgene under the control of the α4 promoter, which is upregulated in epilepsy, was designed to enable self‐regulated modulation of α1 expression. When the AAV was injected in the dentate gyrus (DG), it attenuated acute seizures induced by pilocarpine and significantly reduced the number of animals that developed chronic epilepsy. However, much like the approach targeting NMDA receptors, this strategy resulted in significant side effects, including weight loss and sedation [39].

### Neuropeptides

Neuropeptides represent a crucial target for gene therapy in epilepsy due to their ability to modulate neurotransmission and restore the balance between excitatory and inhibitory neuronal activity. Neuropeptide Y (NPY) has been extensively studied for its anti‐seizure properties and has demonstrated significant potential in preclinical models.

The use of AAV vectors to overexpress NPY has yielded promising results. Early investigations employed recombinant adeno‐associated virus serotype 2 vectors that target the hippocampus [40] or piriform cortex [41] in a rat model of kainate‐induced epilepsy. These investigations demonstrated protective effects, preventing both epilepsy onset and progression to chronic seizures. Interestingly, studies involving NPY Y1 or Y2 receptor (Y2R) double knockout mice revealed that these mice did not benefit from NPY gene therapy, indicating that the activation of one or both receptors is essential for NPY's antiepileptic effects [42].

In a later study, an AAV1/2 vector expressing NPY was administered into the thalamus or somatosensory cortex of a genetic generalized epilepsy rat model, resulting in a reduction in seizure activity, particularly with thalamic injections [43]. However, targeting the hippocampus raises concerns regarding the translational potential of this approach, particularly due to observed impairments in synaptic plasticity and reduced long‐term potentiation at Schaffer collateral‐CA1 synapses following unilateral vector injections [44]. These impairments led to deficits in spatial discrimination learning in naïve rats. Contrary to these findings, a subsequent study demonstrated seizure protection without cognitive impairments in kindled rats that received bilateral injections of the AAV1/2 NPY vector [45].

Despite these encouraging results, early NPY gene therapy studies had limited clinical relevance. These experiments were conducted before the onset of epilepsy, failing to address the pathological changes that occur during epileptogenesis, which could affect treatment efficacy. To overcome this limitation, Noè et al. (2008) examined the effects of hippocampal injections of an AAV1/2 vector expressing NPY after the onset of epilepsy in rats, observing a reduction in seizure activity [46]. This study also found that Y2R levels were maintained in the injected hippocampus, with functional recombinant NPY being released at nerve terminals upon neuronal depolarization [46]. In a follow‐up report, the authors used rAAV1 for NPY delivery, achieving widespread expression throughout the hippocampus and significant seizure reduction without eliciting immune responses or cognitive side effects [47].

Galanin, another neuropeptide with strong therapeutic potential, has also garnered attention for its anti‐seizure and neuroprotective properties. AAV vectors encoding galanin along with a fibronectin secretory signal sequence, designed to facilitate expression and extracellular release, were found to significantly attenuate seizures induced by focal electrical stimulation of the inferior colliculus [48]. Later, the same vector injected into the piriform cortex demonstrated efficacy in reducing seizure sensitivity and delaying seizure progression, with notable neuroprotective effects in models of kainic acid‐induced epilepsy [49]. Here, galanin was also able to prevent kainic acid‐induced hilar neuronal death, highlighting its dual role in mitigating both acute and chronic neurodegeneration associated with epilepsy [49]. Additionally, neuron‐specific enolase‐driven overexpression of galanin in hippocampal neurons was effective in reducing the severity of seizures induced by intrahippocampal kainic acid [50] and excitability in a kindling model, as evidenced by a shortening of afterdischarges [51].

Encapsulated cell biodelivery (ECB) devices represent an innovative alternative for long‐term neuropeptide delivery in epilepsy therapy. These devices encapsulate cells engineered to secrete therapeutic neuropeptides, such as galanin, and have demonstrated promising results in chronic epilepsy models [52]. Unlike direct viral vector delivery, ECB devices mitigate risks associated with genetic modification of host cells, as they enable continuous neuropeptide release without permanent alterations to the patient's genome. Studies using ECB devices in animal models have shown significant reductions in seizure frequency, improved cognitive outcomes, and reversal of histopathological damage associated with chronic epilepsy [52, 53, 54, 55]. Furthermore, the scalability of ECB devices for use in larger animals and early human trials highlights their potential for clinical application, particularly for focal, drug‐resistant epilepsy. A key advantage of ECB devices lies in their tunability, enabling clinicians to precisely adjust neuropeptide release rates to match the severity and frequency of seizures in individual patients. However, for widespread clinical adoption, further optimization is needed to ensure consistent efficacy across diverse patient populations and seizure types.

The therapeutic benefits of neuropeptides extend beyond seizure suppression to encompass neuroprotection and cognitive restoration, an essential consideration given that cognitive decline is a common comorbidity in epilepsy. For instance, overexpression of NPY has been shown to mitigate oxidative stress, reduce neuronal excitotoxicity, and restore cognitive decline in chronic epilepsy models [56]. Similarly, galanin's neuroprotective properties address long‐term neuronal impairment associated with recurrent seizures, emphasizing its utility in neurodegenerative forms of the disease [49]. Interestingly, dynorphins—a related class of neuropeptides—have demonstrated the ability to reverse hippocampal atrophy and cognitive deficits, highlighting the potential of neuropeptides to tackle both symptomatic and structural aspects of chronic epilepsy [57]. Furthermore, behavioral improvements observed in animal studies, such as enhanced memory and reduced aggression, underscore the relevance of neuropeptide therapies in alleviating comorbidities associated with epilepsy [46, 47]. These findings broaden the scope of neuropeptide‐based strategies, highlighting their potential applicability to other neurological conditions, such as Alzheimer's and Parkinson's disease, where excitotoxicity and cognitive decline are key pathological features.

However, it is important to note that some neuropeptide effects—particularly those related to synaptic transmission—were not fully replicated in human epileptic hippocampal tissue. Notably, although NPY influenced glutamate release, galanin failed to produce similar effects in hippocampal neurons [58], underscoring the necessity of translating findings from animal models to human tissue to inform critical decisions and facilitate the advancement of neuropeptide gene therapies into clinical practice. Importantly, NPY was later found to exert significant effects on epileptiform activity in human hippocampal slices [59], further reinforcing its potential as a promising target for clinical translation.

### Growth factors

Along with neuropeptides, growth factors have also been shown to have neuroprotective and anti‐seizure benefits. Brain‐derived neurotrophic factor (BDNF) has gained significant attention for its ability to suppress seizures and address cognitive deficits associated with epilepsy. Research showed that ECB devices, containing genetically modified cells engineered to release BDNF, led to reduced seizure frequency by over 80% in a rat model of chronic TLE [60]. Additionally, ECB delivery demonstrated the ability to restore hippocampal volume and improve neurogenesis, potentially reversing the cognitive impairments often linked to chronic seizures.

The synergistic use of fibroblast growth factor‐2 and BDNF provides an alternative approach to mitigating hippocampal neuronal damage while simultaneously reducing epileptogenesis. Viral vector delivery of these growth factors has been shown to promote neurogenesis by facilitating neural progenitor proliferation and differentiation into functional neurons [61]. In addition, the treatment reduced lesion‐related damage and spontaneous seizures in rodent models of status epilepticus [61]. However, the combined therapy requires further development to ensure sustained and targeted delivery of both factors.

The neuroprotective properties of glial cell line‐derived neurotrophic factor (GDNF) offer another promising pathway for epilepsy therapy. The use of AAV vectors for GDNF delivery in the hippocampus has been shown to reduce seizure frequency and elevate seizure thresholds in preclinical kindling models, highlighting its efficacy in reducing hyperexcitable circuits [62]. Additionally, adenoviral vector‐mediated overexpression of GDNF in the hippocampus could suppress acute tonic–clonic seizures induced by kainic acid [63]. Beyond seizure suppression, GDNF demonstrated the ability to preserve neuronal structures and reduce degeneration, both of which are critical for maintaining neural circuit balance [62]. GDNF showed therapeutic effects also when released from engineered cells in ECB devices, in both kindling [54] and chronic models [53], underscoring its powerful seizure‐suppressant properties in multiple conditions. Recently, GDNF has been shown to mediate an increase in inhibitory synaptic neurotransmission when mouse slices were incubated in vitro [64]. This effect was predominantly postsynaptic and dependent on activation of Ret receptors [64]. Importantly, it was replicated in human epileptic hippocampal slices, suggesting that GDNF might be able to suppress seizures even in the human epileptic hippocampus.

### Adenosine kinase

In addition to targeting ion channels, peptides, and neurotrophic factors, the modulation of adenosine metabolism has also been investigated as a potential therapeutic strategy for epilepsy. Several studies have demonstrated that modulating adenosine kinase (ADK) expression in astrocytes can significantly influence seizure susceptibility. Young et al. explored the downregulation of ADK via miRNA‐mediated gene silencing, reporting a 94%–96% reduction in ADK expression, which led to a 50% decrease in seizure duration in a rat model of TLE, highlighting ADK as a prime therapeutic target [65]. This approach was also validated using AAV8‐mediated antisense RNA expression to selectively suppress ADK in astrocytes, effectively abolishing spontaneous seizures in an epileptic mouse model, reinforcing ADK downregulation as a viable therapeutic strategy [66]. Beyond gene suppression, ex vivo cell‐based adenosine‐releasing grafts have also been explored. When fibroblasts engineered to release adenosine by inhibiting its metabolic enzymes were transplanted into epileptic rats, a near‐complete seizure suppression in the kindling epilepsy model was observed, suggesting that local adenosine delivery can effectively prevent epileptiform activity [67]. Collectively, these findings underscore adenosine‐based gene therapy as a powerful and targeted intervention for drug‐resistant epilepsy, with both viral and cell‐based approaches demonstrating robust preclinical efficacy.

## Advanced gene therapy applications

**Fig. 1:**
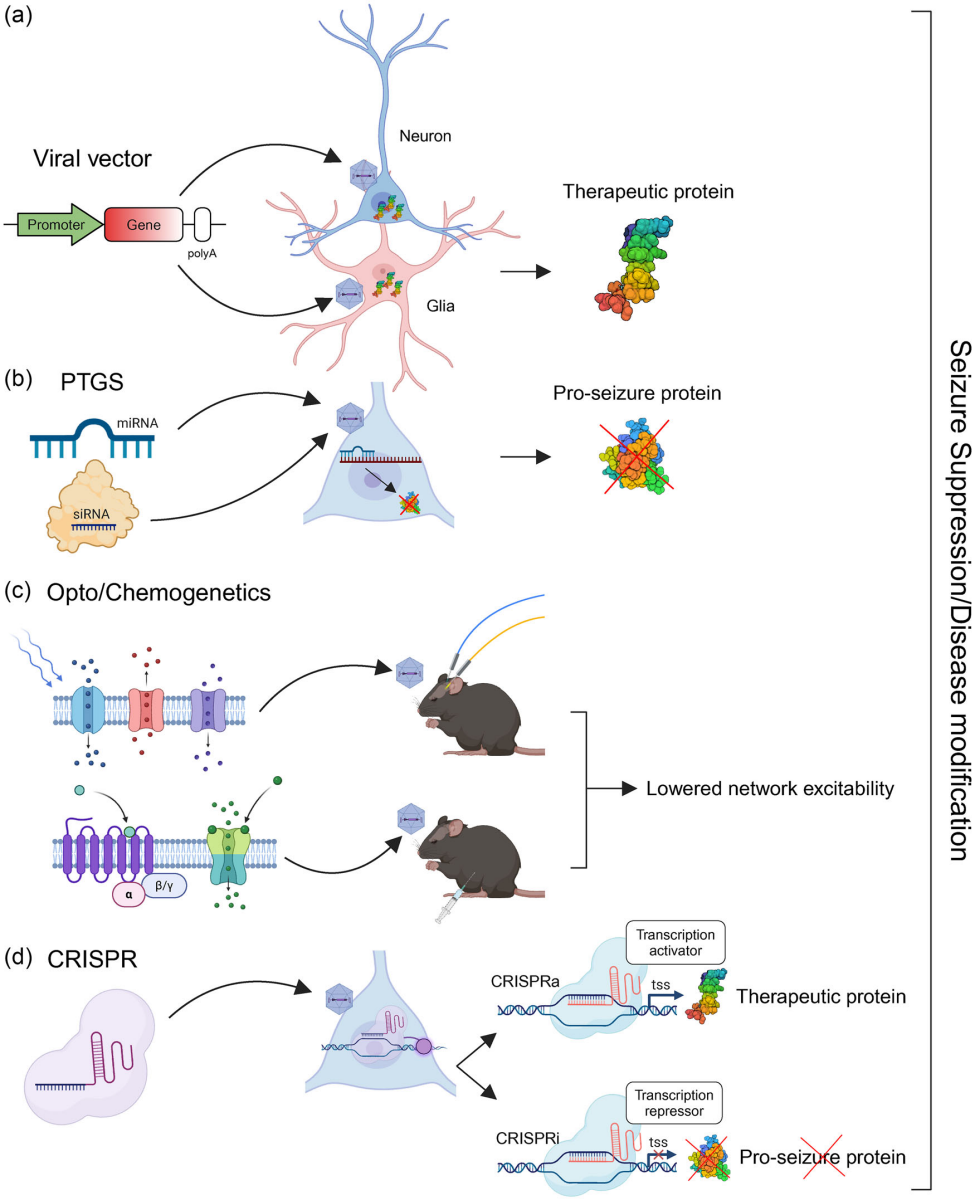
**Summary of gene therapy approaches for seizure suppression and disease modification**. (a) Viral vector mediated gene therapy in neurons or glia for overexpression of therapeutic proteins. (b) Posttranscriptional gene silencing (PTGS), using microRNAs (miRNAs) or siRNAs to block translation of pro‐seizure proteins. (c) Opto‐ and chemo‐genetic approaches for lowering network excitability. (d) Clustered regularly interspaced short palindromic repeat (CRISPR) gene therapy using CRISPRa or CRISPRi to either induce expression of therapeutic proteins or block expression of pro‐seizures targets.

As the field of epilepsy therapy continues to advance, emerging innovative approaches leveraging cutting‐edge gene manipulation techniques are being explored to deliver more precise and effective interventions (Fig. 1). These approaches not only pave the way for more precise treatments but also enhance our understanding of the underlying mechanisms of this complex disorder.

### Optogenetic strategies

Optogenetic strategies employ light‐sensitive proteins to modulate neuronal activity with exceptional temporal and spatial precision. As its introduction, optogenetics has opened new research avenues, significantly advancing both the development of alternative therapeutic strategies and our understanding of physiological and pathophysiological processes in various neurological disorders, including epilepsy and epileptogenesis. Although this review focuses on key strategies that could serve as alternatives for epilepsy gene therapy, we and others have previously provided a comprehensive analysis of how various optogenetic techniques, leveraging the diverse array of light‐sensitive channel variants, have been applied in epilepsy research [68]. For a more in‐depth discussion, we encourage the readers to explore these comprehensive reviews, which provide a foundational understanding of the topics covered in this section.

Broadly, optogenetic strategies for epilepsy treatment operate through one of two core mechanisms: suppressing neuronal excitation or enhancing inhibition. This is achieved by using optogenetic tools, which either hyperpolarize the neuronal membrane (of predominantly excitatory neurons) or depolarize and therefore activate inhibitory neuronal populations.

**Fig. 2:**
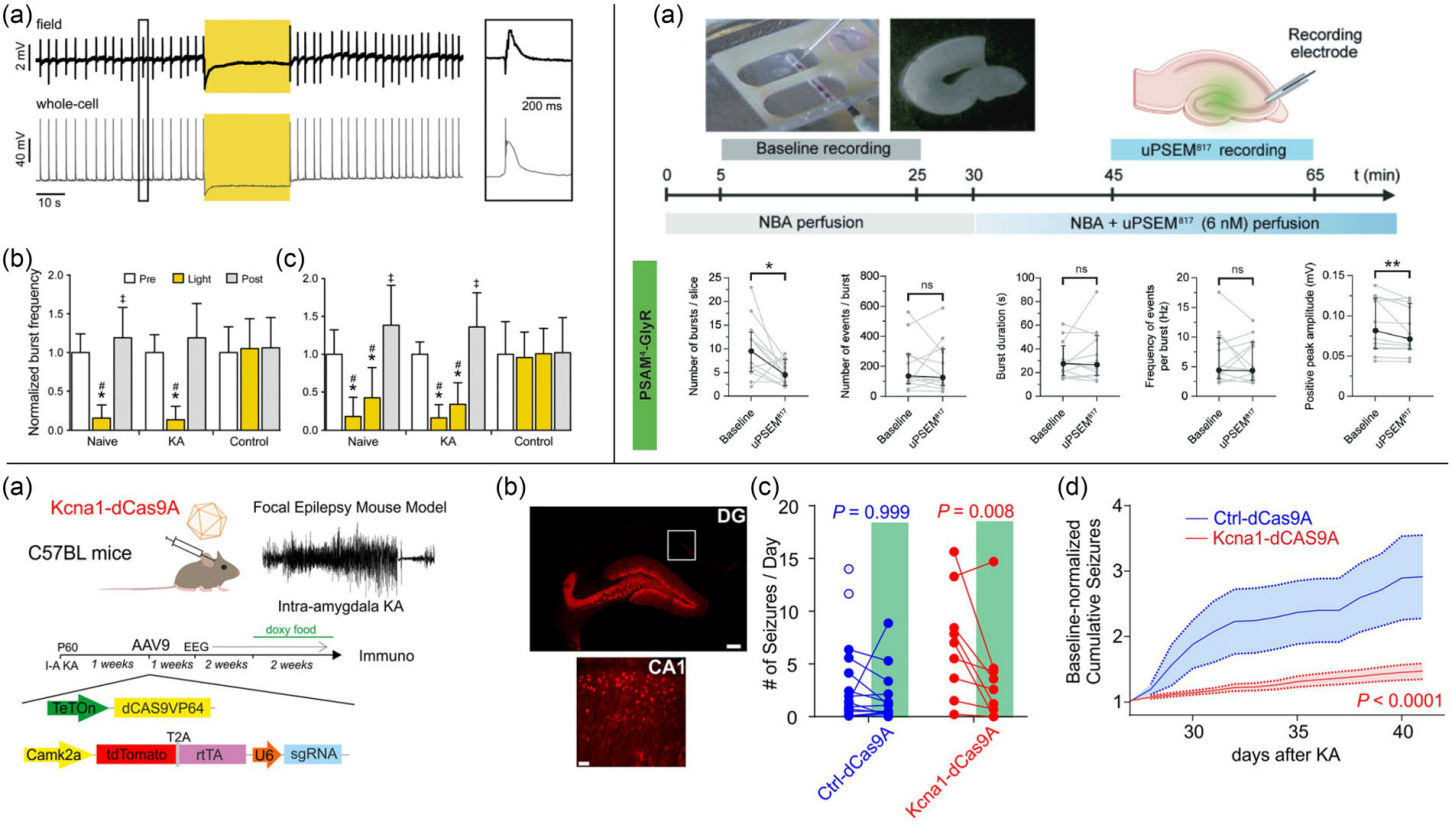
**Optogenetic, chemogenetic, and clustered regularly interspaced short palindromic repeat (CRISPR)/Cas9 gene therapy in epilepsy**. Top left, substantial reduction of PTX‐induced bursting in acute hippocampal slices from NpHR3.0‐expressing mice. (a) Representative recording traces made from the CA3 area of an adeno‐associated virus (AAV)‐NpHR3.0 injected FVB mouse exposed to 30 s 593 nm light (yellow boxes) during a simultaneous field and whole‐cell recording. (b and c) Mean burst frequency of 30 s periods (before, during, and after light) normalized to pre‐light levels, for acute hippocampal slices from NpHR3.0 expressing animals (naïve and KA‐injected) and non‐expressing control animals exposed to 30 s (b) or 60 s (c) of continuous yellow light. Top right, effect of pharmacologically selective effector molecule (uPSEM^817^) on epileptic‐like activity of entorhinal cortex‐hippocampus organotypic slices. (a) Experimental schematic. Above, example images of the recording set‐up, organotypic slices, and the electrode positioning for spontaneous field recordings on CA3. Below, characterization of epileptiform‐like activity and evaluation of the uPSEM^817^ application effect. Scale bar: 200 µm. Bottom, CRISPRa‐Kcna1 decreases the number of seizures in a mouse model of acquired intractable temporal lobe epilepsy. (a) Schematic representation of the CRISPRa approach. (b) Representative immunofluorescence 7 weeks after status epilepticus of neurons transduced with Ctrl‐dCas9A 4 weeks after status epilepticus. Scale bars: dentate gyrus (DG) = 250 µm; CA1 = 50 µm. (c) Number of seizures/day before and after doxycycline administration in control‐dCas9 (n = 13) and Kcna1‐dCas9(n = 9) treated animals. (d) Cumulative plot of seizures normalized to the baseline in mice transduced with either ctrl‐dCas9A orKcna1‐dCas9A. Source: The Figures. modified and reproduced with permission from [30, 70, 108]. NBA, neurobasal A medium.

Among the tools used for inhibition of neuronal activity, the application of halorhodopsin (HR)—a light‐activated chloride pump—has shown significant promise in reducing seizure activity, particularly in rodent models of TLE. Optogenetic inhibition of pyramidal neurons expressing HR could halt excessive synchronized activity induced by stimulation‐induced bursting in vitro [69]. This rapid effect relies on HR's ability to hyperpolarize neurons by increasing intracellular chloride levels, directly counteracting the neuronal hyperexcitability that drives seizure development. The efficacy of this approach was later validated in hyperexcitable slices from epileptic animals [70] (Fig. 2 ) and, most importantly, in vivo across various animal models of focal epilepsy [70, 71].

However, one significant potential drawback of prolonged use of HR is the possible disruption of intracellular chloride homeostasis, which could impact cellular responses, such as GABAergic inhibition. When examining the effects of continuous HR activation for several minutes in CA3 hippocampal pyramidal neurons, we observed not only an increase in depolarizing GABA_A_‐mediated transmission but also an unexpected, significant decrease in action potential threshold [72]. This could potentially increase excitability and counteract the benefits of HR activation for seizure control. Other studies have also reported altered chloride reversal potential following HR activation [73, 74], highlighting the need to carefully consider these consequences when designing experiments. As an alternative, the proton pump ArchT expressed in pyramidal cells has been used to successfully reduce acute neocortical seizures induced by intracortical kainic acid injection [75] and secondary generalized seizures in a kindling model [76]. However, the potential side effects of this approach, such as long‐term changes in extra‐ or intracellular pH, need further evaluation.

Optogenetic applications also extend to network activation, where channelrhodopsin (ChR) variants are employed to depolarize specific neuronal populations. Early pioneering research demonstrated the potential of ChRs as light‐gated cation channels capable of precisely controlling neuronal depolarization [77, 78]. In the context of epilepsy, this mechanism has shown promise in targeting single or multiple populations of inhibitory interneurons to enhance network inhibition and restore the excitatory–inhibitory balance in epileptic circuits by counteracting seizure‐generating activity. Early studies focused on parvalbumin‐ (PV‐) positive interneurons for their ability to effectively inhibit pyramidal cell firing due to the perisomatic location of their efferent synapses. Activation of PV interneurons was effective in reducing both epileptiform activities in vitro [79] and behavioral seizures in vivo [71]. These effects were later replicated by activation of other inhibitory populations, such as somatostatin‐expressing interneurons [79, 80], or by activation of multiple populations simultaneously [76, 79]. Interestingly, in some cases, even activation of pyramidal neurons was able to reduce ictal discharges [80], indicating that the effect of activating neuronal populations on epileptiform activity might be due to perturbation of network synchrony rather than an overall increase in inhibition.

This precise control over neuronal firing offers both therapeutic benefits and critical insights into the pathophysiology of epilepsy. By combining optogenetic interventions with high‐resolution electrophysiological recordings, or even all‐optical experiments with the aid of high‐resolution calcium imaging, researchers can dynamically investigate seizure initiation and propagation mechanisms. This has helped dissect the role of different cell types in ictogenesis, with a prominent example being mossy cells [81]. Although the net excitatory input of mossy cells onto DG granule cells has been previously suggested to be epileptogenic [82, 83], it was their selective optogenetic activation that reduced the duration of electrographic seizures and prevented generalization [81], whereas their inhibition had the opposite effect.

Optogenetics has also shed light on the contribution of distal locations to seizure activity in the epileptic focus. Modulation of cerebellar Purkinje cell activity, or activation of specific cerebellar nuclei, as well as activation of GABAergic neurons in the medial septum, was effective in stopping seizures in the hippocampus of a TLE model [84, 85, 86, 87]. Similarly, modulation of thalamocortical projections was able to suppress seizures in models of experimental stroke [88] and absence seizures [89]. These findings underscore the power of optogenetics in effectively dissecting neuronal networks to precisely interrogate the contribution of specific neuronal ensembles in seizure generation. Even activation of astrocytes by ChR was able to attenuate cortical seizures [75]. However, some research suggests that results obtained from opsin and light‐mediated activation of neurons do not always correspond to responses achieved via more conventional electrical stimulation techniques, underlining the importance of careful experimental planning (including the choice of transgene delivery methods and viral capsids) for a correct interpretation of experimental findings [90].

The incorporation of optogenetics into closed‐loop systems offers an additional dimension of precision and efficiency in managing epilepsy. Closed‐loop paradigms employ real‐time electroencephalographic (EEG) monitoring and seizure‐detecting algorithms that trigger light‐based interventions to suppress seizures [71, 86, 87, 88, 91, 92, 93, 94, 95, 96]. This on‐demand activation minimizes unnecessary stimulation, preserving normal network functionality during periods in between seizures and reducing potential side effects. Moreover, wearable EEG devices and machine learning algorithms are being explored to improve seizure detection accuracy and predict epileptic events before their onset, thereby optimizing the efficacy and timeliness of closed‐loop therapies. These advances align closely with the principles of precision medicine and highlight the potential of optogenetic approaches to transform epilepsy management by offering highly specific and adaptive treatments.

Despite its considerable promise, the translational potential of optogenetics faces significant challenges, particularly regarding safety and delivery. There are potential immunogenic risks associated with the expression of foreign proteins, such as HR or ChRs, which could provoke immune responses and compromise long‐term therapy efficacy. These aspects also raise questions about the correct expression and functionality of optogenetic tools in human tissue. However, viral vectors encoding ChRs have demonstrated successful transduction of hippocampal neurons in cultured hippocampal slices derived from resected epileptic tissue [97]. More recently, the expression of a novel optogenetic potassium channel, HcKCR1 [98], in a subset of excitatory neurons has been shown to effectively reduce epileptiform activity in cultured human tissue [99]. These data help bridge the gap between rodent and human studies, paving the way for further clinical translation.

In conclusion, optogenetics offers a groundbreaking approach to epilepsy therapy, providing unparalleled temporal and cell‐specific precision in modulating neuronal activity and targeting epilepsy‐relevant circuits. Although the preclinical evidence showcases its immense potential, significant challenges remain in translating these findings into clinical practice. The integration of optogenetics with advanced delivery methods and closed‐loop systems offers a promising pathway to overcome these hurdles, ultimately enhancing its applicability and efficacy for individuals with epilepsy. Continued research and innovation in this field are essential for unlocking the full potential of optogenetics in revolutionizing epilepsy treatment.

### Chemogenetics

Chemogenetics utilizes genetically engineered receptors—such as designer receptors exclusively activated by designer drugs (DREADDs) and engineered ligand‐gated ion channels—to externally modulate neuronal excitability in a controlled manner [100, 101]. Similarly to optogenetics, these approaches allowed the design of experiments that have offered invaluable insight into pathophysiological network function mechanisms and expanded into alternative therapies for several neurological disorders. Here, we will focus on some of the recent research using chemogenetic tools for the treatment of epilepsy.

Several studies have explored region‐specific chemogenetic silencing to control epilepsy. hM4Di DREADDs were employed to silence pyramidal neurons in the subiculum, a hyperexcitable region in TLE, and demonstrated a robust reduction in both acute and chronic seizures in rodent models [102]. Similarly, the hippocampal CA3 region and the anterior nucleus of the thalamus were targeted using hM4Di‐expressing AAVs [103]. Their results indicated a significant reduction in seizure frequency and duration following ligand administration, emphasizing the potential of remote modulation of seizure networks [103].

Beyond seizure suppression, chemogenetics has been investigated for its effects on epilepsy‐associated cognitive deficits. Yang et al. demonstrated that chemogenetic inhibition of subicular seizure‐activated neurons alleviated cognitive impairments in a mouse model of TLE. Their study highlighted the importance of selectively targeting CaMKIIα+ excitatory neurons while preserving GABAergic interneurons, as nonspecific inhibition could impair cognitive functions [104].

The DG plays a critical role in gating excessive excitatory input to the hippocampus. By chemogenetically increasing dentate granule cell (DGC) excitability, it was found that excessive excitation of DGCs alone was sufficient to induce seizures in non‐epileptic mice and impair spatial memory [105]. These results validate the dentate gate hypothesis, suggesting that chemogenetic restoration of DG inhibition may be a viable treatment for epilepsy, thereby cementing earlier findings with optogenetics [93].

The application of chemogenetics was also extended to nonhuman primates to assess its feasibility as a clinically relevant approach for seizure suppression [106]. This study focused on the attenuation of cortical seizures using engineered receptors that allowed for precise and reversible neuronal inhibition. By employing hM4Di DREADDs targeted to excitatory neurons, the researchers demonstrated that systemic administration of the selective ligand effectively suppressed seizure activity without inducing significant behavioral side effects. Importantly, their findings showed that chemogenetic intervention reduced both seizure duration and frequency, offering strong preclinical support for the translational potential of this technology. By demonstrating successful modulation of seizure dynamics in a primate model, this research paves the way for future clinical applications and underscores chemogenetics as a promising, minimally invasive therapeutic strategy for epilepsy management in humans [106].

Other chemogenetic tools, such as Pharmacologically Selective Actuator Modules (PSAMs), have also been used in epilepsy research. The PSAM4‐glycine receptor (GlyR) system consists of a chimeric receptor combining the ligand‐binding domain of the α7 nicotinic acetylcholine receptor (α7‐nAChR) with the anion‐permeable pore domain of a GlyR. The receptor is specifically activated by a pharmacologically selective effector molecule (uPSEM^817^), a synthetic ligand that is inert under normal physiological conditions but effectively opens the channel when applied. This activation permits chloride influx, leading to neuronal hyperpolarization and inhibition of excitability [107]. Unlike DREADDs, which rely on G‐protein‐coupled receptor signaling and can exhibit variability in effectiveness due to downstream pathway alterations in epilepsy, the PSAM4‐GlyR system offers a direct and reliable means of modulating membrane potential. Moreover, the ligand uPSEM^817^ does not interact with endogenous receptors, reducing off‐target effects and making the system highly specific.

We investigated the potential of PSAM4‐GlyR to suppress neuronal excitability and epileptiform activity. The study aimed to evaluate whether chemogenetic modulation of inhibitory ion channels could provide an effective strategy for seizure control. Neurons expressing PSAM4‐GlyR exhibited a significant decrease in excitability following uPSEM^817^ application, as evidenced by a reduction in input resistance and action potential frequency. In ex vivo epilepsy models using hippocampal slices, the system demonstrated a 47% reduction in spontaneous burst frequency and decreased peak amplitude of epileptiform discharges. This indicates that PSAM4‐GlyR activation effectively inhibits hyperexcitable circuits in epileptic tissue [108] (Fig. 2 ).

The study highlighted the potential of PSAM4‐GlyR as an alternative to DREADD‐based approaches, offering a more direct, ionotropic mechanism for neuronal inhibition. However, challenges remain in achieving optimal expression, refining ligand delivery, and enhancing overall efficacy for seizure suppression. Despite these limitations, PSAM4‐GlyR‐based chemogenetics represents a promising avenue for targeted, noninvasive epilepsy therapy. Importantly, PSAM4‐GlyR receptors are also readily activated by varenicline, a drug utilized for smoking cessation, at nanomolar concentrations, making this tool particularly attractive for clinical scenarios.

Another similar tool is the bradanicline and ACh‐activated receptor for neuronal inhibition (BARNI), which integrates the α7‐nAChR ligand‐binding domain with an α1 GlyR anion pore domain. When activated by bradanicline—a clinically evaluated α7 nAChR agonist—BARNI effectively suppressed neuronal activity and reduced seizure frequency in mice [109]. Notably, endogenous ACh levels rose sharply during seizures, suggesting that BARNI may also function in an on‐demand manner, potentially reducing the need for continuous drug administration.

Despite promising results, several challenges remain. Many current chemogenetic systems (DREADDs in particular) rely on ligands such as clozapine‐*N*‐oxide (CNO), which can be converted to clozapine, a compound with off‐target effects. The development of clinically viable ligands such as bradanicline and uPSEM^817^ represents a significant step forward. Achieving long‐term, cell‐type‐specific expression while minimizing immune responses and off‐target effects remains a significant challenge. Although nonhuman primate studies are encouraging, additional clinical trials are needed to evaluate safety, efficacy, and long‐term effects. The ability of BARNI to respond to endogenous ACh surges offers a compelling on‐demand seizure control mechanism, but concerns related to ligand specificity, delivery methods, and translation to humans must be addressed before chemogenetics can become a mainstream clinical therapy.

### CRISPR‐based engineering

As its discovery as part of the adaptive immune system of several bacteria and archaea species, the CRISPRs system has been quickly adapted as a powerful tool for genome engineering in mammalian cells. The editing system relies on three main components: a CRISPR‐associated (Cas) endonuclease, a guide RNA (gRNA) to direct the Cas protein to the desired genomic location, and a protospacer adjacent motif (PAM) sequence on the genome, required for Cas activity [110]. The system is easily programmable in the laboratory to target virtually any genomic location by simply designing specific 20‐nucleotide sequences of the gRNA, complementary to target regions in the genome immediately ahead of a Cas‐specific PAM sequence. Once delivered to the target cells, the Cas/gRNA ribonucleoprotein (RNP) complex binds the target genomic DNA, and the endonuclease introduces breaks in the sequence immediately ahead of the PAM sequence. The type of genomic edit that follows depends on endogenous cellular repair pathways: homology‐dependent repair, which uses a DNA template to repair breaks, or nonhomologous end joining (NHEJ), which rapidly ligates DNA without the need for templates and often results in insertion or deletion of base‐pairs at the break site. The type of repair is largely dependent on the cell type targeted, with postmitotic cells—such as neurons—often preferring NHEJ. Because of these properties, CRISPR/Cas has most often been used to disrupt protein function, following indel formation in coding exons to generate frameshift or introduce stop codons. However, to our knowledge, gene disruption by CRISPR has still not been used in the context of epilepsy as a therapeutic strategy. One theoretical possibility could be to target single alleles in conditions where gain‐of‐function of the *SCN1A* [111] or *SCN8A* [112] genes is linked to epileptic encephalopathies.

The recent expansion of the CRISPR toolbox with the addition of base editors—utilizing a Cas nickase to generate single‐strand breaks in combination with a base deaminase [113, 114, 115]—enables the correction of single‐point mutations. Although this approach has not yet been used in neurons, once optimized, its application for epilepsy therapy could potentially be invaluable, as at least 4000 single‐nucleotide mutations leading to pathologic epileptic phenotypes have already been discovered (NCBI ClinVar database).

Other compelling alternatives to irreversible CRISPR/Cas‐mediated gene editing leverage the system's specificity to reversibly modulate gene expression instead, thus offering greater flexibility and control in therapeutic applications. By utilizing a deactivated Cas protein (dCas) and fusing it with transcriptional activators or repressors, it is possible to target regulatory regions of genes of interest and either increase or suppress their expression [116]. These alternative uses of CRISPR—termed CRISPRa (activation) and CRISPRi (interference), respectively—could in some cases be more desirable than permanent editing solutions and increase safety profiles.

For epilepsy gene therapy, one notable application is the use of CRISPRa to enhance the expression of Kv1.1 potassium channels, which play a critical role in stabilizing neuronal excitability. Increased Kv1.1 expression through CRISPRa significantly reduces seizure frequency in TLE models [30] (Fig. 2). Similarly, CRISPRa‐mediated upregulation of Nav1.1 sodium channels in inhibitory GABAergic neurons has shown efficacy in improving both survival rates and seizure outcomes in mouse models of Dravet syndrome [34]. By leveraging endogenous gene loci, CRISPRa offers an advantage over traditional methods of upregulation, as it minimizes risks associated with excessive therapeutic protein levels, enhancing safety. The level of gene upregulation can be modulated by choosing weaker or stronger transcriptional activators—such as VP64, VPR, or synergistic activation mediators—or by utilizing multiple gRNAs to align cellular responses with the desired amount of overexpression [116, 117].

CRISPRi complements CRISPRa by selectively silencing genes implicated in the epileptic phenotype, providing a precise and adaptable approach to gene regulation in epilepsy therapy. The dCas9–KRAB–MeCP2 system has demonstrated robust transcriptional inhibition in neurons, offering improved precision compared to traditional RNA interference methods [118]. For instance, silencing synaptotagmin I (Syt1), a gene essential for excitatory neurotransmitter release, has been particularly effective in modifying the excitatory–inhibitory balance within the DG of rodent models. This method might help to address hyperexcitability in epileptogenic circuits by targeting specific neuronal populations [118]. Other cases where CRISPRi might be implemented include genetic epilepsies arising from gain‐of‐function mutations, such as some epileptic encephalopathies (SCN2A, SCN8A, or KCNT1 mutations) or focal cortical dysplasia where the mTOR pathway is upregulated. Designing targeted CRISPRi therapies to downregulate key components of the mTOR pathway could provide a promising alternative to current pharmacological treatments with mTOR inhibitors [119]. This approach offers enhanced specificity while minimizing side effects associated with systemic drug administration.

**Fig. 3:**
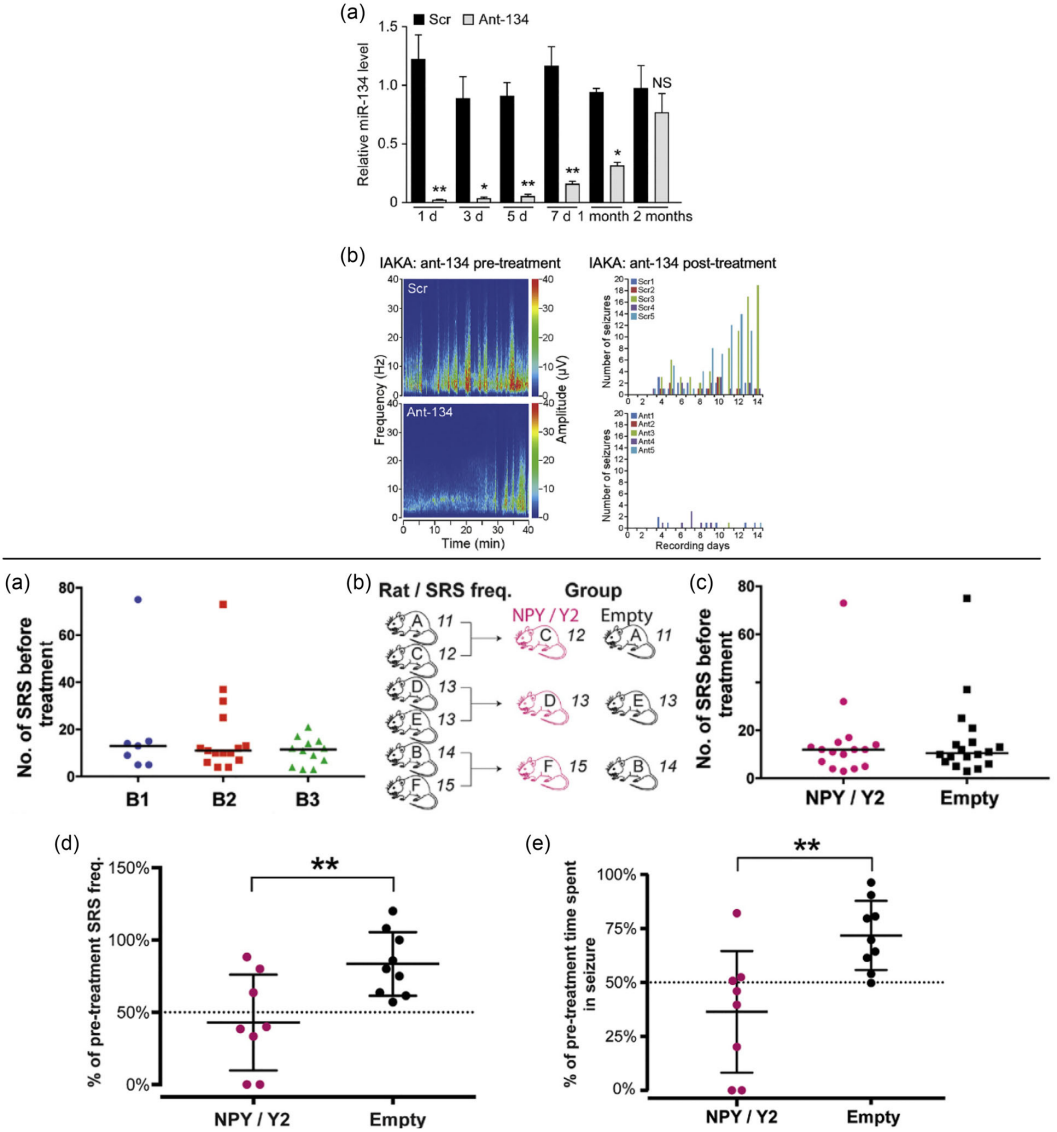
**Gene therapy for epilepsy using** microRNAs **(miRNAs), antagomirs, and combinatorial approaches**. Top left, miR‐134 knockdown is therapeutic in various in vivo experimental models and species. (a) In vivo intracerebroventricular injection of ant‐134 in mice mediates a significant knockdown of miR‐134 for one month. (b) IAKA—intra‐amygdalakainic acid: Spectrograms show that status epilepticus reduced by pretreatment with ant‐134 (left panel). Posttreatment with ant‐134 leads to a reduction in spontaneous seizures during the chronic phase of this model (right panel). Bottom, combinatorial gene therapy with adeno‐associated virus (AAV) expressing neuropeptide Y (NPY) and Y2 receptors. (a) The number of SRS during the pretreatment monitoring period was found not to differ between the groups, indicating equal disease severity across the three batches. (b) The animals were assigned to the treatment groups with the aim of stratifying them for equal mean SRS frequency. (C) No difference in SRS frequency during 3 weeks of monitoring before treatment indicated a successful stratification of the groups. Bottom right, (d) the average decrease in SRS frequency in nonprogressive animals was greater in the NPY/Y2‐treated group compared to control animals. (e) The nonprogressive animals in the NPY/Y2‐treated group also displayed a significantly greater reduction in total time spent in seizure compared to controls. Source: The figures. modified and reproduced with permission from [130, 133]. IAKA, intra‐amygdalakainic acid.

An alternative approach to gene suppression in epilepsy involves the use of miRNAs, small noncoding RNAs that regulate gene expression at the posttranscriptional level. miRNAs function by binding to complementary sequences in the 3′ untranslated region of target mRNAs, leading to mRNA degradation or translational repression. Dysregulated miRNA networks have been implicated in seizure pathophysiology, making them an attractive therapeutic target for controlling epilepsy‐related gene expression. Delivery of an miRNA responsible for the regulation of several voltage‐gated sodium channels via AAV9 (miR‐335‐5p) in the hippocampus was recently shown to effectively suppress seizures and increase survival in the model of acute seizures [120]. Similarly, downregulation of miR‐134 by specific antagomirs after the induction of SE significantly reduced the number of seizures and, in addition, protected against progressive TLE pathology, as shown by a better‐preserved number of hippocampal neurons after treatment [121, 133] (Fig. 3 ).

Although miRNA‐based therapies offer advantages—such as leveraging endogenous regulatory pathways—they also face limitations, including off‐target effects, transient activity, and the need for repeated dosing. Unlike CRISPRi, which enables sustained gene regulation, miRNA therapies require ongoing intervention, presenting a significant challenge for long‐term treatment strategies. Furthermore, CRISPRi and CRISPRa are both capable of multiplex gene repression, enabling the simultaneous targeting of multiple genes implicated in seizure activity. This flexibility makes them particularly valuable in cases where intervention on single genes is not sufficient to provide adequate symptomatic control or disease modification.

Despite the promise of CRISPR systems, delivery challenges remain a major obstacle to their application in epilepsy. The small payload capacity of AAVs limits their compatibility with large CRISPR systems, necessitating innovations such as alternative or miniaturized Cas enzymes [122] or other delivery vectors if large brain areas are to be covered, as in the case of genetic epilepsies. For NHEJ‐based approaches requiring a single administration of the system, direct delivery of the Cas/gRNA RNPs complex could represent a valid alternative, as it has already been shown to successfully mediate editing in neurons [123, 124].

The integration of CRISPR systems with advanced technologies such as optogenetics and chemogenetics offers exciting possibilities for further improving therapeutic precision. Light‐inducible CRISPR systems combine the spatial precision of optogenetics with the genetic specificity of CRISPR, enabling real‐time disruption of epileptogenic circuits. For instance, light‐activated transcriptional modulation could allow researchers to temporally align therapeutic interventions with seizure onset, or perhaps with circadian rhythms, reducing unnecessary gene modulation and enhancing treatment efficacy [125]. Similarly, drug‐activated CRISPR modalities incorporate chemogenetic control [126, 127], providing tunable and on‐demand therapeutic options for seizure management. By transforming static systems into dynamic, adaptive platforms, these approaches promise to open new avenues in precision medicine. However, their clinical implementation still requires overcoming challenges related to delivery efficiency and long‐term therapeutic stability.

Long‐term safety and potential adverse effects represent a critical challenge. Off‐target effects, which may disrupt genomic stability or induce unwanted alterations of expression in unrelated genes, remain a primary concern. Advanced gRNA design algorithms and high‐fidelity Cas variants have been developed to mitigate this issue, but off‐target effects remain a possibility that needs to be carefully controlled. Potential immunogenicity of long‐term expression of Cas proteins also presents a barrier to clinical translation, even though recent data show that Cas9 RNPs are minimally immunogenic [123].

In conclusion, CRISPR‐based gene editing presents a highly promising therapeutic avenue for epilepsy, with applications ranging from gene modulation to precise genetic repair. Innovative delivery systems and the integration of complementary technologies such as optogenetics and chemogenetics hold the potential to overcome current limitations, paving the way for personalized and dynamic therapeutic interventions in epilepsy. Continued interdisciplinary research is essential to address safety, scalability, and adaptability challenges, ultimately unlocking the full potential of CRISPR technologies in revolutionizing epilepsy treatment.

### Combinatorial gene therapy

Epilepsies are complex disorders, and in some cases, single‐target gene therapy might not be able to offer sufficient symptomatic relief or significant disease modification. In addition, the effect of overexpression of ligands such as NPY or GDNF might be constrained by the limited availability of receptors necessary to mediate the desired biological effects. To address these limitations, researchers have investigated combinatorial gene therapy, a strategy where two or more therapeutic genes are used simultaneously to enhance synergistic effects and improve treatment efficacy.

Early key evidence supporting the use of combinatorial gene therapy for epilepsy came from studies that independently delivered NPY and its Y2R using two distinct AAV vectors [128]. This approach demonstrated that overexpression of Y2R alone in the hippocampus significantly reduced seizure activity in rodent models of TLE. However, they also observed that the combined overexpression of both NPY and Y2R led to a more pronounced seizure‐suppressant effect, albeit using two separate vectors [128].

This study was foundational but also highlighted potential limitations in using dual‐vector systems. The separate administration of AAV‐Y2 and AAV‐NPY introduced variability in gene expression levels and distribution, which could affect therapeutic consistency. Recognizing these challenges, subsequent research efforts focused on optimizing this approach by combining both genes into a single AAV vector. Later studies refined this strategy by engineering a single AAV construct encoding both NPY and Y2R within the same expression cassette. We were among the first to demonstrate that a single AAV1 vector carrying NPY and Y2R, arranged in a bicistronic expression system (NPY‐IRES‐Y2 or Y2‐IRES‐NPY), resulted in superior and more stable transgene expression compared to separate vectors (Fig. 3). Our findings indicated that the AAV1 serotype with the NPY‐IRES‐Y2 arrangement provided optimal seizure suppression and significantly reduced glutamate release in epileptic hippocampal tissue [129, 130]. Szczygieł et al. further validated this approach in preclinical studies, demonstrating that a single‐vector system (CG01) encoding both NPY and Y2R maintained stable long‐term expression without adverse effects on cognition or body weight [131]. Meanwhile, others explored a lentiviral approach targeting DG granule cells. Unlike AAV vectors, which have a broader distribution, LVs allow for more precise targeting of excitatory neurons. This study also introduced an autoregulatory mechanism in which NPY and Y2 expression were controlled by a minimal CamKIIα promoter. The advantage of this approach is that it enables self‐regulation of neuronal excitability, ensuring that inhibition only occurs when neuronal activity becomes excessive. This approach showed a significant reduction of seizure frequency and duration in the synapsin triple knockout model [132].

The transition from separate AAV vectors for NPY and Y2R to a single‐vector system represents a crucial step toward clinical translation. The single‐vector approach offers improved consistency, reduces the complexity of administration, and ensures co‐expression of both therapeutic genes within the same neurons, enhancing the overall efficacy of gene therapy for epilepsy.

Taken together, these studies highlight the potential of combinatorial gene therapy in epilepsy. The evidence strongly suggests that targeting both NPY and Y2 can produce robust seizure suppression, making this a highly promising avenue for clinical translation. Future research will need to address optimal dosing, safety in humans, and the feasibility of large‐scale vector production to pave the way for clinical trials. Combinatorial gene therapy targeting NPY and Y2 is a groundbreaking approach for treating drug‐resistant epilepsy. The research to date has consistently demonstrated that AAV‐ and LV‐mediated overexpression of these genes reduces seizure activity through mechanisms involving glutamate release inhibition and enhanced neuronal regulation. Although challenges remain in translating these findings into clinical applications, the preclinical data are highly encouraging and suggest that NPY gene therapy could soon become a viable alternative for epilepsy patients.

## Clinical trials and current status

### Gene therapy

Several clinical trials are currently underway, focusing on different gene therapy strategies to address various forms of epilepsy, particularly Mesial Temporal Lobe Epilepsy and Dravet Syndrome (Table 1). These trials explore the use of viral vectors to deliver therapeutic genes aimed at reducing seizure frequency and improving neurological function.

**Table 1:** Clinical trials in gene and cell therapy for epilepsy.

NCT number	Title	Status	Condition	Intervention	Start date	Primary completion	Completion	URL
Gene therapy								
NCT06706388	Personalized Antisense Oligonucleotide Therapy for a Single Participant with ATN1 Gene Mutation	ACTIVE, NOT RECRUITING	Dentatorubral–Pallidoluysian Atrophy	DRUG: nL‐ATN1‐002	2024‐02‐21	2026‐02	2026‐02	https://clinicaltrials.gov/study/NCT06706388
NCT06112275	A Clinical Study to Evaluate the Safety and Efficacy of ETX101, an AAV9‐Delivered Gene Therapy in Children With SCN1A‐positive Dravet Syndrome (Australia Only)	RECRUITING	Dravet Syndrome	DRUG: ETX101 (AAV9)	2024‐02‐28	2029‐12	2029‐12	https://clinicaltrials.gov/study/NCT06112275
NCT06283212	A Clinical Study to Evaluate the Safety and Efficacy of ETX101, an AAV9‐Delivered Gene Therapy in Children With SCN1A‐positive Dravet Syndrome	RECRUITING	Dravet Syndrome	DRUG: ETX101 (AAV9)	2024‐05‐09	2029‐12	2029‐12	https://clinicaltrials.gov/study/NCT06283212
NCT05419492	A Clinical Study to Evaluate the Safety and Efficacy of ETX101 in Infants and Children with SCN1A‐Positive Dravet Syndrome	RECRUITING	Dravet Syndrome	DRUG: ETX101 (AAV9)	2024‐05‐14	2027‐04	2031‐04	https://clinicaltrials.gov/study/NCT05419492
NCT06063850	AMT‐260 Gene Therapy Study in Adults with Unilateral Refractory Mesial Temporal Lobe Epilepsy	RECRUITING	Mesial Temporal Lobe Epilepsy	GENETIC: AAV9‐hSyn1‐miGRIK2	2024‐06	2026‐11	2027‐06	https://clinicaltrials.gov/study/NCT06063850
NCT04601974	Lentiviral Gene Therapy for Epilepsy	NOT YET RECRUITING	Drug Resistant Epilepsy	GENETIC: lentiviral gene therapy	2024‐09	2028‐09	2032‐09	https://clinicaltrials.gov/study/NCT04601974
NCT07102524	Intrathecal Gene Therapy For SLC13A5 Citrate Transporter Disorder (SLC13A5)	NOT YET RECRUITING	SLC13A5 Citrate Transporter Disorder	DRUG: TSHA‐105	2025‐12‐01	2030‐12‐01	2031‐06‐01	https://clinicaltrials.gov/study/NCT07102524
Cell therapy
NCT06638970	Clinical Utility and Safety of Human Umbilical Cord Mesenchymal Stem Cell Secretome in Drug Resistant Epilepsy Single Center, Non‐randomized, Phase I Clinical Trial	NOT YET RECRUITING	Epilepsy, Drug Resistant Epilepsy	DRUG: Secretome	2024‐12	2025‐10	2026‐10	https://clinicaltrials.gov/study/NCT06638970
NCT06422923	A Phase 1/2 Study of NRTX‐1001 Neuronal Cell Therapy in Drug‐Resistant Bilateral Mesial Temporal Lobe Epilepsy (MTLE)	RECRUITING	Epilepsy, Temporal Lobe	BIOLOGICAL: NRTX‐1001	2024‐11‐14	2026‐07‐15	2040‐07‐15	https://clinicaltrials.gov/study/NCT06422923
NCT05135091	FIH Study of NRTX‐1001 Neural Cell Therapy in Drug‐Resistant Unilateral Mesial Temporal Lobe Epilepsy	RECRUITING	Mesial Temporal Lobe Epilepsy With Hippocampal Sclerosis	BIOLOGICAL: NRTX‐1001 PROCEDURE: Sham Comparator	2022‐06‐16	2025‐05	2026‐05	https://clinicaltrials.gov/study/NCT05135091
NCT05886205	Induced Pluripotent Stem Cell Derived Exosomes Nasal Drops for the Treatment of Refractory Focal Epilepsy	RECRUITING	Refractory Focal Epilepsy	DRUG: iPSC‐Exos	2023‐06‐05	2025‐06‐13	2025‐11‐13	https://clinicaltrials.gov/study/NCT05886205

Abbreviations: AAV, adeno‐associated virus; FIH, first‐in‐human; iPSC, induced pluripotent stem cell.

One of the ongoing studies, AMT‐260, is investigating gene therapy for adults with unilateral mesial temporal lobe epilepsy. This study employs the AAV9‐hSyn1‐miGRIK2 vector, which is designed to modulate glutamate receptor function to achieve seizure suppression. The primary objective of this study is to evaluate the safety and tolerability of the treatment while monitoring its potential efficacy. With an expected completion date in 2027, this study represents one of the most advanced efforts to apply gene therapy for focal epilepsy.

For genetic epilepsies such as Dravet Syndrome, multiple trials are focusing on the use of ETX101, a gene therapy‐based drug designed to correct underlying molecular defects. The WAYFINDER study is a phase 1/2 trial currently recruiting patients in Australia, aiming to assess the safety and preliminary efficacy of ETX101. Similarly, the EXPEDITION trial, which is United Kingdom‐based, is evaluating the same intervention in a different population. Both studies are scheduled for completion in 2029 and are critical for establishing gene therapy as a viable treatment for Dravet Syndrome.

Another major effort, the ENDEAVOR study, is investigating gene therapy for Dravet Syndrome in a more extensive and long‐term framework. This trial is designed as a multiphase study that evaluates the impact of ETX101 on seizure control and neurological development. Expected to continue until 2031, it represents one of the most comprehensive studies in gene therapy for epilepsy. By focusing on genetic correction mechanisms, these trials are exploring novel ways to provide long‐lasting therapeutic benefits to individuals with Dravet Syndrome.

These ongoing clinical trials demonstrate a growing confidence in gene therapy as a transformative approach for epilepsy treatment. By utilizing viral vectors to deliver precise genetic modifications, these studies aim to correct or compensate for underlying molecular dysfunctions that contribute to epileptic activity. The success of these trials could pave the way for a new era in epilepsy management, offering hope to patients who have not responded to traditional medications. Although safety remains a primary concern in the early phases, these studies are setting the foundation for the eventual widespread application of gene therapy in neurological disorders.

### Cell therapy

Several clinical trials are currently underway, focusing on different cellular interventions to modulate neural activity and reduce seizure occurrence (Table 1). These trials explore the use of stem cell‐derived therapies and neuronal cell transplantation to restore normal brain function and provide long‐term benefits for epilepsy patients.

One ongoing study investigates the safety of autologous mesenchymal stem cell (MSC) infusion for epilepsy treatment. This trial aims to evaluate the safety and feasibility of using a patient's own MSCs to reduce seizure frequency and improve overall neurological health. The findings from this study will contribute to the growing evidence that MSCs may have neuroprotective and anti‐inflammatory properties beneficial for epilepsy management.

Another major study currently recruiting patients is a phase 1/2 trial exploring NRTX‐1001, a neuronal cell therapy for TLE. This study is designed as a multicenter, single‐arm, open‐label investigation to assess the safety and preliminary efficacy of NRTX‐1001 transplantation in patients with focal epilepsy. The therapy involves the transplantation of human stem cell‐derived inhibitory neurons into the hippocampus, with the goal of restoring inhibitory balance and reducing seizure activity. A related first‐in‐human study is also underway to evaluate the effectiveness of NRTX‐1001 specifically in patients with mesial TLE with hippocampal sclerosis. This trial follows a similar design but includes a sham comparator to rigorously assess the impact of the treatment.

Another innovative approach being investigated is the use of induced pluripotent stem cell (iPSC)‐derived exosomes for refractory focal epilepsy. This study is evaluating the safety, tolerability, and preliminary efficacy of iPSC‐derived exosomes as a novel therapeutic modality for difficult‐to‐treat epilepsy cases. Exosomes are small vesicles released by cells that can carry bioactive molecules, including proteins and RNA, which may have therapeutic effects by modulating inflammation and neuronal excitability.

The ongoing clinical trials in cell therapy for epilepsy underscore the growing interest in regenerative medicine as a novel treatment strategy for drug‐resistant epilepsy. Unlike traditional therapies that primarily focus on symptom suppression, these approaches aim to modify neural circuitry by introducing new, functional cells or bioactive molecules. Stem cell‐derived neurons have the potential to integrate into existing networks, restoring inhibitory control and reducing hyperexcitability associated with seizures. Similarly, exosome‐based therapies offer a noninvasive way to deliver therapeutic molecules that can modulate inflammation and neuronal activity. While still in the early phases of clinical research, these cell‐based interventions hold significant promise for offering long‐lasting improvements in seizure control and neural function. If proven safe and effective, they could redefine the future of epilepsy treatment by addressing the underlying causes of the disorder rather than just managing its symptoms.

## Challenges and future directions

Gene therapy for epilepsy has shown significant promise in preclinical and early clinical trials, particularly for drug‐resistant cases. However, several challenges remain before this approach can become a mainstream treatment. One of the major hurdles is the delivery method. AAV vectors, which are commonly used in gene therapy, have limitations in terms of their capacity to carry therapeutic genes and their ability to achieve widespread transduction in the brain without invasive administration. The blood–brain barrier poses an additional obstacle, making it difficult to ensure efficient and widespread gene delivery to affected regions. Another challenge is long‐term gene expression. Although sustained therapeutic effects are desirable, prolonged overexpression of certain genes could lead to tolerance or unexpected side effects. More advanced gene regulation systems—such as inducible promoters or controlled expression through CRISPR‐based gene modulation—will be necessary to fine‐tune the level of therapeutic gene expression over time.

A significant concern with gene therapy is off‐target effects, where unintended genetic modulation may occur in nontarget tissues or cell types, leading to unforeseen neurological or systemic consequences. Ensuring precise targeting while maintaining a broad enough distribution to be effective requires careful vector design and delivery strategies. Additionally, immune responses to viral vectors or introduced gene products can hinder treatment efficacy and pose safety risks. Developing stealthier vector systems, immune‐modulating therapies, or alternative delivery mechanisms such as lipid‐based nanoparticles may help circumvent these issues. Personalized approaches are another crucial aspect of gene therapy that needs further refinement. Epilepsy is a highly heterogeneous condition, with multiple underlying genetic and environmental factors influencing disease progression and treatment response. A one‐size‐fits‐all approach is unlikely to be effective, making it imperative to develop individualized gene therapy strategies based on a patient's specific epilepsy etiology and genetic profile. Finally, regulatory challenges pose a major barrier to clinical translation. Gene therapy is still an evolving field, and different countries have varying regulations regarding its approval and commercialization. Standardizing protocols, ensuring comprehensive long‐term safety data, and streamlining the regulatory approval process will be key to making gene therapy widely accessible.

Cell therapy possesses its own set of challenges that must be addressed before it can be widely implemented. Unlike gene therapy, which primarily aims to modify existing neuronal activity, cell therapy involves introducing new cells into the brain to restore lost or dysfunctional inhibitory function. One of the fundamental issues is ensuring that transplanted cells integrate properly into the host brain without causing adverse effects such as tumor formation, immune rejection, or excessive inhibition leading to cognitive impairments. The choice of cell type is also crucial, as different types of stem cell‐derived neurons may have varying capacities to form appropriate synaptic connections and regulate seizures effectively. Current clinical trials have demonstrated the feasibility of stem cell‐derived inhibitory neuron transplantation, but these studies are still in early phases, with limited long‐term data on safety and durability of effects. Another major challenge is the survival and functional integration of transplanted cells. The brain's microenvironment presents difficulties in ensuring that transplanted neurons establish lasting and effective connections without disrupting existing neural circuits. Optimizing the microenvironment through supportive factors such as neurotrophic signals, biomaterial scaffolds, or co‐transplantation with glial cells may improve the long‐term success of cell therapies.

Regulatory and ethical considerations further complicate the clinical implementation of cell therapy. The production of high‐quality, standardized neural cell lines requires rigorous oversight to ensure consistency, safety, and effectiveness. In particular, the use of human‐derived pluripotent stem cells raises ethical concerns regarding donor consent, genetic modifications, and potential misuse of the technology. Additionally, like gene therapy, cell‐based therapies face regulatory challenges in different regions, requiring harmonization of approval processes and long‐term monitoring of patients to assess efficacy and safety. Cost and scalability are also major hurdles; producing patient‐specific or off‐the‐shelf cell therapies at a scale suitable for widespread clinical use remains technically and financially challenging. Future advancements in biomanufacturing, automation, and cell engineering techniques may help address these issues, but significant investment and collaboration between academia, industry, and regulatory agencies will be necessary to bring these therapies to the clinic.

Both gene and cell therapies for epilepsy hold immense potential, but their successful clinical translation will require a concerted effort to refine delivery mechanisms, improve safety profiles, and establish standardized protocols for widespread use. Future research must prioritize strategies that balance efficacy with minimal invasiveness, ensuring that these therapies are not only effective but also practical and accessible to patients. Advances in biomaterials, precision medicine, and artificial intelligence‐driven optimization of treatment protocols could accelerate the progress of these therapies, making them viable alternatives to conventional pharmacological and surgical treatments. Addressing the regulatory and logistical barriers will be critical in determining whether these approaches can move beyond experimental treatments into mainstream clinical practice, offering new hope for epilepsy patients who have exhausted all other options.

## Conflict of interest statement

The authors declare no conflicts of interest.
